# Mechanisms and active components of *Solanum nigrum* in the amelioration of psoriatic lesions

**DOI:** 10.3389/fimmu.2026.1801799

**Published:** 2026-04-24

**Authors:** Ya Chen, Tianyou Ma, Congcong Zhu, Zongguang Tai, Huijun Pan, Zhongjian Chen, Quangang Zhu

**Affiliations:** 1 Shanghai Skin Disease Hospital, School of Medicine, Tongji University, Shanghai, China; 2 Shanghai Engineering Research Center for Topical Chinese Medicine, Shanghai, China

**Keywords:** NLRP3 inflammasome, pattern recognition receptors, psoriasis, *Solanum nigrum*, trigonelline

## Abstract

**Introduction:**

Psoriasis is a prevalent chronic inflammatory skin disease in which pattern recognition receptors, particularly the NLRP3 inflammasome, are increasingly implicated in disease pathogenesis. Solanum nigrum (SN) has been used in traditional and clinical practice for psoriasis treatment, but its therapeutic mechanisms and key active constituents remain unclear. This study investigated the anti-psoriatic mechanisms of SN and identified its major bioactive component.

**Methods:**

NLRP3 inflammasome activation in psoriasis was evaluated using public transcriptomic datasets and clinical skin biopsies. The therapeutic effects of SN were assessed in imiquimod-induced primary and relapse psoriasis-like dermatitis models. Bulk RNA sequencing of lesional skin was performed to identify SN-regulated pathways. SN was chemically characterized by UPLC–MS, and candidate active compounds were prioritized by molecular docking and molecular dynamics simulation.

**Results:**

NLRP3 inflammasome activation was consistently elevated in psoriatic lesions in both public datasets and clinical specimens. SN markedly alleviated disease severity in primary and relapse models, reduced keratinocyte hyperproliferation, and lowered systemic inflammatory cytokine levels. Transcriptomic analysis showed that SN mainly modulated PRR/NLR-related signaling pathways. Mechanistically, SN inhibited NLRP3 inflammasome activation and decreased IL-1β and IL-18 production. Integrated chemical, biological, and computational analyses identified trigonelline as a major active constituent contributing to the anti-psoriatic effects of SN.

**Discussion:**

SN ameliorates psoriasis-like dermatitis primarily through suppression of NLRP3 inflammasome signaling, with trigonelline identified as a key contributory active component. These findings provide mechanistic support for the therapeutic application of SN in psoriasis.

## Graphical Abstract

**Figure:**
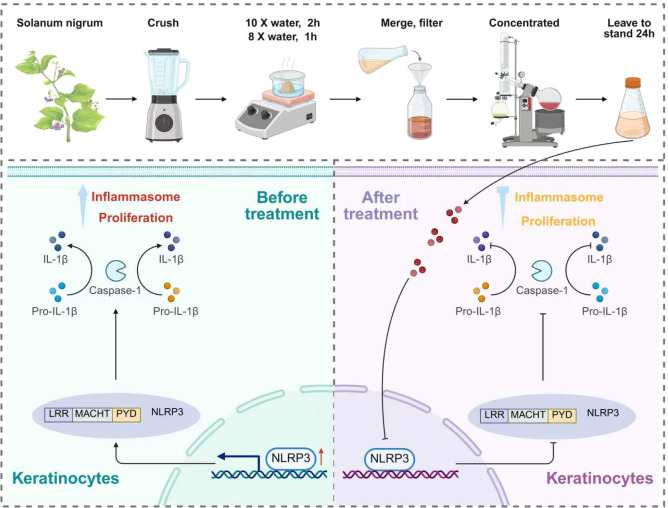

## Introduction

Psoriasis is a chronic inflammatory skin disorder characterized by excessive proliferation and aberrant differentiation of keratinocytes, accompanied by prominent immune cell infiltration (1). Its pathophysiology involves a complex interplay among genetic susceptibility, environmental triggers, and immune dysregulation (2). Among the immune pathways implicated in psoriasis, the interleukin-23 (IL-23)/T helper 17 (Th17) axis is widely recognized as a central driver of disease initiation and progression (3). This pathway coordinates the activation of multiple immune cell subsets and inflammatory mediators, thereby sustaining chronic cutaneous inflammation. In addition to adaptive immune responses, increasing evidence indicates that innate immune signaling mediated by pattern recognition receptors (PRRs) also plays a critical role in the inflammatory cascade of psoriasis (4). Notably, nucleotide-binding oligomerization domain-like receptor protein 3 (NLRP3), an important member of the PRR family, has been reported to be aberrantly activated in psoriatic lesions (5).

The NLRP3 inflammasome is a multiprotein complex composed of NLRP3, apoptosis-associated speck-like protein containing a caspase recruitment domain (ASC), and pro-Caspase-1 (6). As an intracellular PRR complex, the NLRP3 inflammasome senses pathogen-associated molecular patterns (PAMPs) and damage-associated molecular patterns (DAMPs), thereby initiating innate immune responses and modulating downstream adaptive immunity. Upon activation, NLRP3 promotes Caspase-1 cleavage and activation, which subsequently drives the maturation and secretion of pro-inflammatory cytokines such as interleukin-1β (IL-1β) and interleukin-18 (IL-18) (7). IL-1β binds to its receptors on dermal T cells, promoting their proliferation and stimulating IL-17 production, which further activates keratinocytes to release chemokines and perpetuate local inflammation (8). Meanwhile, IL-18 can synergize with IL-23 to amplify inflammatory signaling and contribute to psoriasis-like epidermal hyperplasia (9).

Consistent with this mechanism, aberrant activation of the NLRP3 inflammasome has been documented in both clinical samples and experimental models of psoriasis. Su et al. reported NLRP3 expression in psoriatic samples was fourfold higher than in normal skin biopsy samples (10). Aira et al. observed increased expression of Caspase-1, IL-1β, and IL-18 in lesional skin compared with non-lesional skin biopsies from psoriasis patients (11). These observations suggest that the NLRP3 inflammasome contributes to psoriatic inflammation and represents a potential therapeutic target.

*Solanum nigrum* (SN) is a widely distributed medicinal herb that has been used for inflammatory dermatoses for centuries. In clinical practice, fresh SN leaves are commonly crushed and applied topically to relieve inflammation and treat skin disorders, while decoctions prepared from the whole plant are administered orally for their detoxifying and anti-inflammatory effects (12). Modern pharmacological studies have demonstrated that SN possesses anti-inflammatory activity, which has been largely attributed to its alkaloid constituents (13). Several alkaloids identified in SN, including solasonine, solamargine, and trigonelline, have been reported to modulate inflammatory signaling pathways and immune responses (14, 15).

Despite its long history of clinical use, the active components and molecular mechanisms underlying the anti-psoriatic effects of SN remain insufficiently defined. Notably, SN is a key herb in the clinically used Longkui Yinxiao formulation at Shanghai Skin Disease Hospital and is characterized by stable supply, low cost, and a chemically defined alkaloid-rich profile, enabling mechanistic deconvolution and quality-control development (16). Given the central role of inflammasome activation in psoriasis pathogenesis, we hypothesized that SN exerts therapeutic effects by suppressing NLRP3 inflammasome signaling. To test this hypothesis, we integrated analyses of public transcriptomic datasets and clinical biopsies, primary and relapse psoriasis-like mouse models, transcriptomic profiling of lesional skin, UPLC–MS-based chemical characterization, and molecular docking together with molecular dynamics simulation to delineate a targetable mechanism and prioritize key bioactive components.

## Materials and methods

### Reagents and antibodies

SN was obtained from Shanghai Cai Tong De Tang Traditional Chinese Medicine Pharmaceutical Factory Co., Ltd. (Shanghai, China). Imiquimod (IMQ) cream (H20100157, Aldara, Leicestershire, UK) was used to establish the psoriasis-like model. All primers were synthesized by Sangon Biotech (Shanghai, China). Primary antibodies against NLRP3 (30109-1-AP), IL-1β (16806-1-AP), and Caspase-1 (31020-1-AP) were obtained from Proteintech Inc. (Chicago, USA), while IL-18 (A20473) was purchased from Abclonal Inc. (Hubei, China). Ki-67 rabbit monoclonal antibody (AF1738), PCNA rabbit monoclonal antibody (AF1363), HRP-conjugated goat anti-rabbit IgG (H+L) (A0208), and HRP-conjugated goat anti-mouse IgG (H+L) (A0216) were obtained from Beyotime Biotechnology (Shanghai, China). Enhanced chemiluminescence (ECL) reagents were purchased from Yeasen (Shanghai, China).

### Animals and treatment

Male BALB/c mice (6–8 weeks old) were obtained from Sipeifu (Suzhou) Biotechnology Co., Ltd. and maintained under standard specific pathogen-free conditions (22–25 °C, 30–70% relative humidity, 12 h light/dark cycle) with free access to food and water. After one week of acclimatization, dorsal hair was removed before model induction.

For the primary model, mice were randomly assigned to five groups: Control, Vehicle-IMQ, IMQ + SN-Low, IMQ + SN-High, and IMQ + MTX. Psoriasis-like dermatitis was induced by topical application of 5% imiquimod (IMQ) cream (Aldara; H20100157, Leicestershire, UK) to the shaved dorsal skin (approximately 2 cm × 3 cm) once daily for 14 consecutive days at 62.5 mg per mouse per day, except in the Control group. The Control group received vehicle treatment only. SN was administered by oral gavage 6 h after IMQ application at low and high doses corresponding to 1× and 4× the clinical equivalent dose, respectively, and methotrexate (MTX) (Sphsine; H31020644, Shanghai, China) was used as a positive control. Dose conversion and preparation are described in the **Supplementary Material**. The Vehicle-IMQ group received the same IMQ induction with vehicle administration. Clinical severity was evaluated daily using a modified Psoriasis Area and Severity Index (PASI) based on erythema, scaling, and thickness, each scored from 0 to 4, giving a total score of 0–12. Skin thickness was additionally measured using a digital caliper.

For the relapse model, mice were grouped as Non-relapse, Relapse-IMQ, Relapse + SN-Low, and Relapse + SN-High. After the initial induction/treatment phase, mice in the relapse groups underwent a 21-day recovery period and were then re-challenged on the same dorsal site with IMQ at 31.25 mg per mouse per day for 7 consecutive days. The Non-relapse group did not receive IMQ re-challenge. Lesion scoring, histopathological evaluation, and immunohistochemical quantification were performed in a blinded manner. For terminal procedures, mice were anesthetized with chloral hydrate according to the approved animal protocol; blood was collected by retro-orbital sampling, and lesional skin and spleen tissues were harvested. Animals were euthanized under deep anesthesia in accordance with institutional animal welfare requirements and relevant veterinary guidance (including AVMA recommendations). All procedures were approved by the Ethics Committee of Shanghai Skin Disease Hospital (Approval No.: 2025-10[animal]). All animal groups and interventions are listed in **Supplementary Table 1**.

### Preparation of SN

SN was decocted to prepare an aqueous extract. Briefly, dried SN was boiled in 10 volumes (v/w) of purified water for 2 h, followed by a second decoction in 8 volumes of water for 1 h. Filtrates were combined, concentrated to a crude-drug equivalent concentration of ~0.216 g/mL, and allowed to stand for 24 h. The supernatant was collected and filtered prior to use. Dose calculations and crude-drug equivalent conversions are provided in the **Supplementary Material**.

### The analysis of clinical dataset

Public psoriasis transcriptomic datasets were retrieved from the Gene Expression Omnibus (GEO). GSE13355 and GSE14905 were selected because they included lesional skin from patients with psoriasis and healthy control skin, had adequate sample sizes, and contained probes for NLRP3 inflammasome-related genes. Expression matrices and phenotype data were downloaded and grouped according to the original annotations. Differential expression analysis was performed using the limma package in R. When raw data were available, background correction and quantile normalization were applied. Genes with an adjusted P value < 0.05 after Benjamini-Hochberg correction were considered significantly differentially expressed. Expression levels of NLRP3, CASP1, IL18, and IL1B were visualized using boxplots with individual data points.

### Histopathological analysis

Dorsal skin tissues were fixed in 10% buffered formalin, embedded in paraffin, sectioned at 4–5 μm, and stained with hematoxylin and eosin (H&E). For each animal, three non-consecutive sections were analyzed, and the mean value was used for statistical analysis. Sections were examined under a Nikon Eclipse E100 light microscope. Histopathological features, including epidermal hyperplasia, parakeratosis, and dermal inflammatory cell infiltration, were evaluated in a blinded manner.

### Human skin biopsy specimens

Lesional skin biopsies from patients with psoriasis vulgaris (n = 7) and normal skin samples from healthy controls (n = 7) were collected for immunohistochemistry. Participants were enrolled according to the ethics approval described in the Clinical sample section. No systemic immunosuppressive therapy was administered within 4 weeks prior to biopsy. All samples were processed in parallel, and investigators were blinded to group identity during IHC scoring.

### Immunohistochemistry staining

Paraffin-embedded sections were deparaffinized, rehydrated, subjected to antigen retrieval, and blocked with 5% bovine serum albumin for 20 min at room temperature. Sections were incubated overnight at 4 °C with primary antibodies against PCNA, Ki-67, NLRP3, IL-18, Caspase-1, and IL-1β, followed by incubation with HRP-conjugated secondary antibodies and chromogenic detection. For each sample, three non-consecutive sections were stained, and three non-overlapping high-power fields were captured per section (200×). Staining was semi-quantitatively scored based on staining intensity and the percentage of positive cells (score range: 0–12), as detailed in the **Supplementary Material**. All sections were independently evaluated by two investigators blinded to group allocation, and the mean score was used for analysis.

### Enzyme-linked immunosorbent assay

Serum was collected by centrifugation at 12,000 × g, 4 °C for 20 min. Levels of IL-1β (EM30300S), TNF-α (EM30536S), IFN-γ (EM30253S), and IL-17A (EM30282S) were measured using commercial ELISA kits (Biotechwell, Shanghai, China) following the manufacturers’ protocols.

### RNA sequencing

Dorsal skin tissues from the Control, IMQ, and IMQ-SN-high groups were collected on day 14 for bulk RNA sequencing (n = 4 per group). Total RNA was extracted using TRIzol reagent, and RNA quality was assessed before library preparation. Libraries were prepared using the Illumina TruSeq Stranded mRNA Kit and sequenced on an Illumina platform. Raw reads were quality-checked with FastQC and processed to remove low-quality reads and adapters. Clean reads were aligned to the reference genome using HISAT2, and transcript abundance was quantified with StringTie. Differentially expressed genes were identified using a standard RNA-seq analysis pipeline with adjusted P < 0.05 as the significance threshold. Gene Ontology (GO) and Kyoto Encyclopedia of Genes and Genomes (KEGG) enrichment analyses were subsequently performed to identify significantly altered biological processes and pathways.

### Molecular docking

2D ligand structures were retrieved from PubChem, converted to mol2 format via Chem3D 19.0, and prepared as pdbqt files using AutoDockTools 1.5.7. Target protein structures were obtained from the Protein Data Bank (PDB), and hydrogens were added. Docking simulations were performed using AutoDock Vina, and results were visualized with PyMOL 3.2.

### Western blotting

Skin tissues (30 mg) were homogenized in ice-cold RIPA buffer containing protease and phosphatase inhibitors. Lysates were incubated on ice for 10 min and centrifuged at 12,000 × g for 15 min at 4 °C. Supernatants were collected and protein concentrations were measured using a BCA assay. Equal amounts of protein (20 μg) were separated by SDS-PAGE (10–12% gels) and transferred onto PVDF membranes. After blocking with 5% non-fat milk in TBST for 1 h at room temperature, membranes were incubated with primary antibodies overnight at 4 °C, followed by HRP-conjugated secondary antibodies for 1 h at room temperature. Signals were detected using ECL reagents and quantified by densitometry with ImageJ. Protein expression levels were normalized to β-actin.

### Real- time PCR

Total RNA was extracted using Novizan Vazyme reagent (RMA101-C2-P1) and reverse-transcribed into cDNA using HiScript III RT premix (R323-0). Quantitative PCR was performed with ChamQ Universal SYBR qPCR reagent (Q711-03) on a LightCycler480 system. Primer sequences are listed in **Supplementary Table 2**.

### UPLC/MS analysis

Chemical profiling of the aqueous SN extract was performed using an ACQUITY UPLC I-Class HF system coupled to a Q Exactive Orbitrap mass spectrometer. For sample preparation, 100 μL extract was mixed with methanol containing internal standard, vortexed, ultrasonicated in an ice-water bath, and centrifuged. The supernatant was collected, diluted as required, and transferred to LC-MS vials for analysis.

Chromatographic separation was achieved on an ACQUITY UPLC HSS T3 column (100 mm × 2.1 mm, 1.8 μm) at 45 °C using water containing 0.1% formic acid as mobile phase A and acetonitrile as mobile phase B. MS data were acquired in both positive and negative ion modes using a heated electrospray ionization source. Full MS and data-dependent MS/MS spectra were collected over the m/z range of 100–1200.

Raw data were processed using Progenesis QI v3.0. Feature annotation was performed based on accurate mass, retention behavior, and MS/MS fragmentation by comparison with the Traditional Chinese Medicine database and manual curation using extracted ion chromatograms. Pooled quality-control samples and internal standards were used to monitor instrument stability and analytical reproducibility. Detailed chromatographic gradients and MS parameters are provided in the **Supplementary Material**.

### Molecular dynamics simulation

Protein-ligand complexes were simulated using GROMACS 2025 for 100 ns, including energy minimization, NVT, and NPT equilibration. Structural stability was assessed via RMSD, residue flexibility via RMSF, molecular compactness via radius of gyration (Rg), solvent exposure via SASA, and hydrogen bond dynamics. Binding free energy and residue decomposition were calculated using the MM-PBSA method.

### Statistical analysis

Statistical analyses were performed using GraphPad Prism 9.0 and R (version 4.3.2). Data are presented as mean ± SD. Comparisons among multiple groups were performed using one-way ANOVA followed by Tukey’s *post hoc* test, unless otherwise stated. For GEO transcriptomic analyses, differential expression was performed using limma with Benjamini-Hochberg false discovery rate correction. A two-sided P < 0.05 was considered statistically significant. The specific sample size and statistical methods used for each experiment are indicated in the corresponding figure legends.

## Results

### NLRP3 inflammasome is overactivated in psoriatic lesions

**Figure 1:**
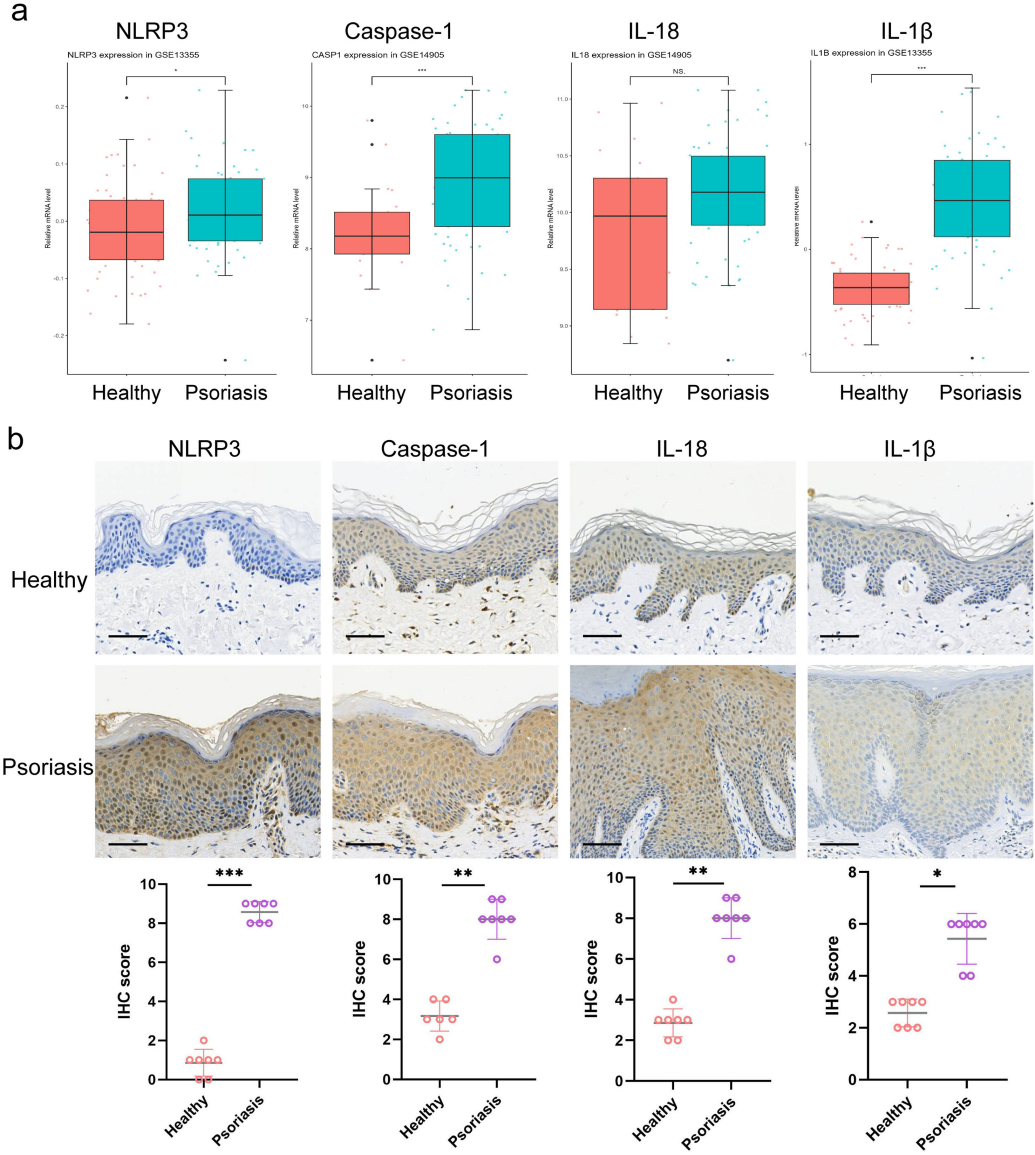
NLRP3 inflammasome is overactivated in psoriatic lesions. **(a)** Transcriptomic analysis of NLRP3, Caspase-1, IL-18 and IL-1β expression in healthy skin and psoriasis skin based on the GSE13355 and GSE14905; **(b)** IHC images of NLRP3, Caspase-1, IL-18 and IL-1β in normal skin from healthy controls and lesional skin tissue from psoriasis patients (200×). n=7 samples/group and data are presented as mean ± SD. ***p < 0.001, **p < 0.01, *p < 0.05 vs. the Healthy group; ns means no significance. Scale bar = 100 μm.

The NLRP3 inflammasome is a multiprotein complex that functions as a critical component of innate immunity (17). To evaluate its involvement in psoriasis, we analyzed two publicly available transcriptomic datasets (GSE13355 and GSE14905) and examined the expression of NLRP3 inflammasome-related genes, including CASP1, IL18, and IL1B. Compared with healthy skin, psoriatic lesions exhibited significantly increased expression of NLRP3, IL18, and IL1B (**Figure 1a**), whereas CASP1 mRNA showed no significant difference. Given that inflammasome activation is largely regulated by post-translational Caspase-1 cleavage rather than transcript abundance, we next validated the pathway at the protein level. Immunohistochemistry in clinical biopsies and murine psoriasiform skin demonstrated increased protein expression of NLRP3 and associated components (Caspase-1, IL-1β, and IL-18) relative to controls (**Figure 1b**). Consistently, in the IMQ-induced model, lesional skin displayed elevated mRNA expression of NLRP3, Caspase-1, IL-1β, and IL-18. Collectively, these data indicate aberrant activation of the NLRP3 inflammasome in both human psoriatic lesions and murine psoriasis-like skin.

### SN alleviates skin lesions in IMQ-induced psoriasiform mice

**Figure 2:**
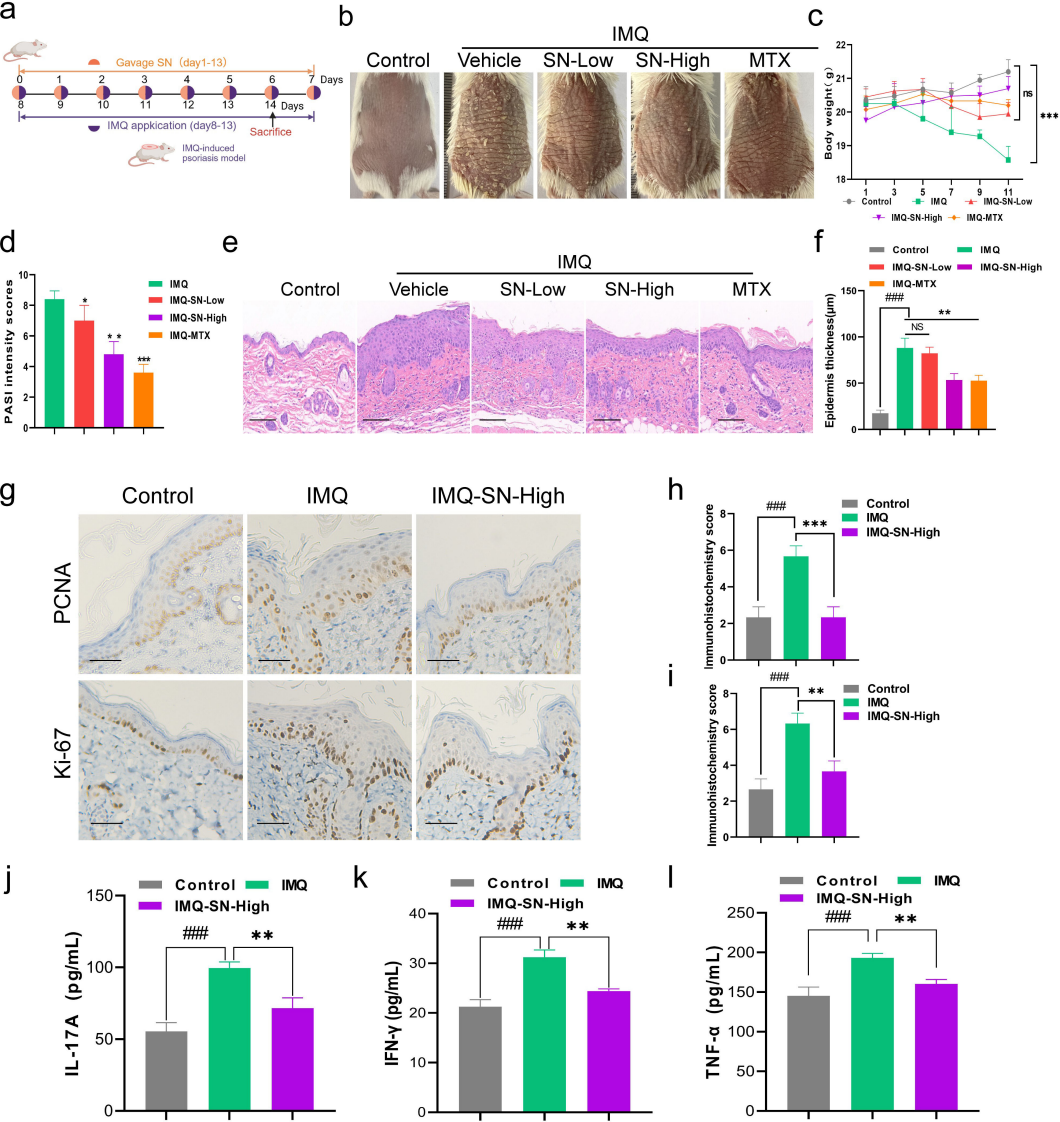
SN ameliorates psoriasiform skin lesions and inhibits inflammatory responses *in vivo*. **(a)** Experimental design of IMQ-stimulated psoriasis-like mouse model; **(b)** Improvement of psoriasiform skin lesions by SN (n=4 samples/group); **(c)** Effect of SN on body weight in psoriasiform mice; **(d)** Impact of SN on PASI scores in psoriasiform mice; **(e, f)** Histopathological morphology of dorsal skin tissue, epidermal thickness, and statistical analysis (×200); **(g-i)** IHC detection and statistical analysis of PCNA and Ki-67 expression in skin lesions (×200); **(j-l)** Effect of SN on inflammatory cytokines in psoriasiform mice. data are presented as mean ± SD. ###p < 0.001 vs. the Control group; *p < 0.05, **p < 0.01, and ***p < 0.001 vs. the IMQ group. Scale bar = 100 μm.

Low-dose and high-dose SN were administered at 1× and 4× the clinical equivalent dosage, respectively. The experimental design is illustrated in **Figure 2a**. IMQ application induced pronounced erythema and scaling on the dorsal skin, closely resembling the clinical manifestations of psoriasis, whereas SN treatment markedly ameliorated these pathological features (**Figure 2b**). Consistently, both low- and high-dose SN significantly reduced Psoriasis Area and Severity Index (PASI) scores in a dose-dependent manner (**Figure 2d**). Body weight analysis revealed a significant reduction in the IMQ-treated group (P < 0.0001), while no significant weight loss was observed in SN-treated mice (P > 0.05), indicating a favorable safety profile (**Figure 2c**). Histopathological examination using hematoxylin and eosin (H&E) staining demonstrated severe epidermal disruption in IMQ-treated mice, characterized by loss of the granular layer, parakeratosis, elongated rete ridges, dilated capillaries, and prominent inflammatory cell infiltration in the dermis. In contrast, mice receiving high-dose SN exhibited a largely restored epidermal architecture, reduced parakeratosis, and significantly decreased epidermal thickness (**Figures 2e, f**). Immunohistochemical analysis further showed that the proliferation markers Ki-67 and proliferating cell nuclear antigen (PCNA) were markedly upregulated in the IMQ group but were significantly downregulated following SN treatment (**Figures 2g–i**), suggesting effective suppression of keratinocyte hyperproliferation. Moreover, serum levels of pro-inflammatory cytokines, including TNF-α, IL-17A, and IFN-γ, were significantly reduced in SN-treated mice (**Figures 2j–l**), further supporting its anti-inflammatory activity. Collectively, these results demonstrate that SN effectively alleviates IMQ-induced psoriasiform skin lesions while exhibiting a favorable safety profile.

### SN prevents psoriasis relapse

**Figure 3:**
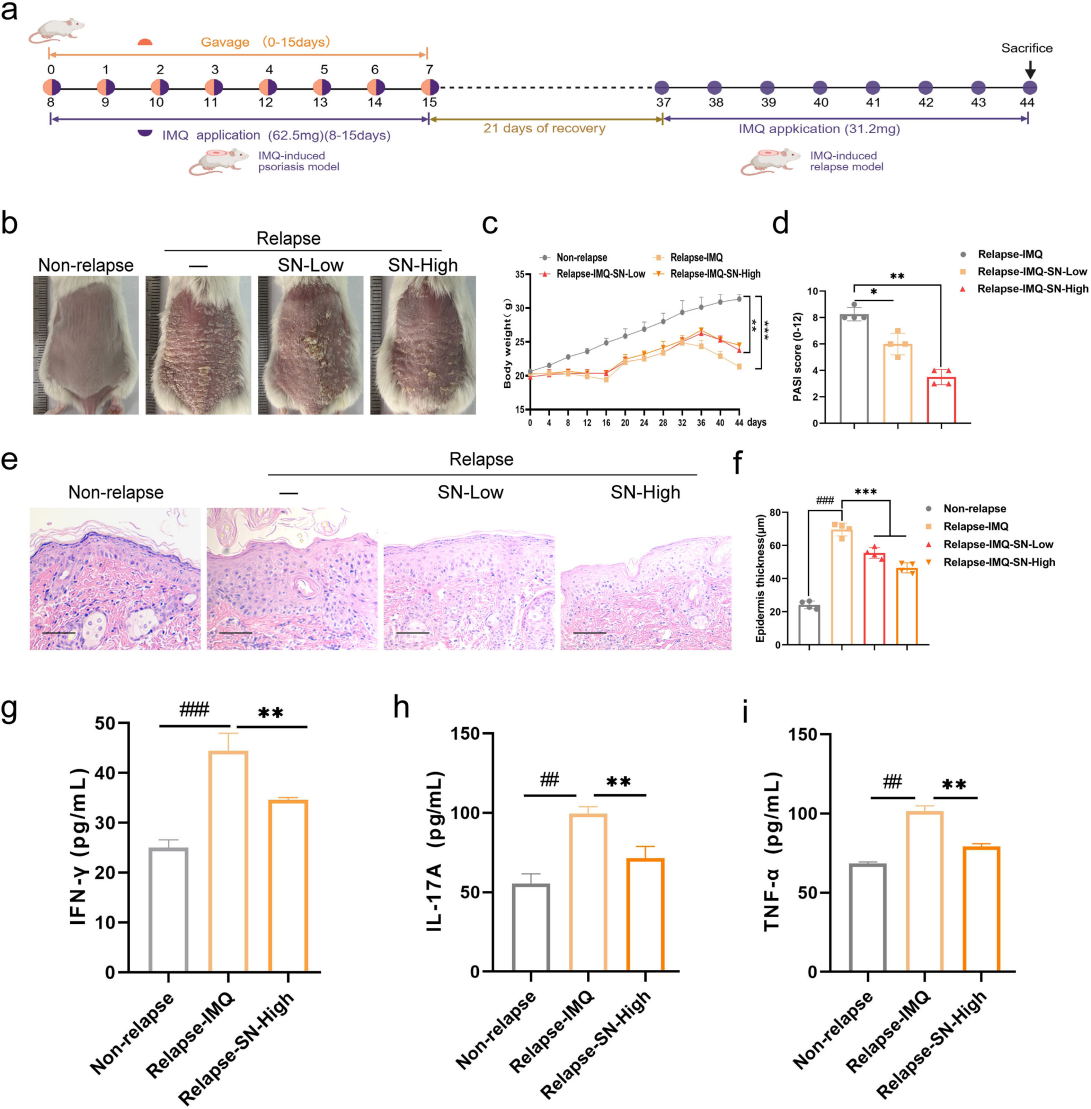
SN suppresses psoriasis-like relapse after IMQ re-challenge. **(a)** Experimental design of IMQ-stimulated psoriasis relapse model; **(b)** Representative images of mice in the relapse model; **(c, d)** Quantifications of body weight **(c)** and PASI score **(d)**; **(e, f)** H&E staining of dorsal skin sections **(e)** and quantification of epidermal thickness **(f)**; **(g-i)** Serum levels of inflammatory cytokines in the relapse model. data are presented as mean ± SD (n = 4). ###p < 0.001, ##p < 0.01 vs. the Non-relapse group; *p < 0.05, **p < 0.01, and ***p < 0.001 vs. the Relapse-IMQ group. Scale bar = 100 μm.

To evaluate whether prior SN treatment confers sustained protection against secondary IMQ challenge, a psoriasis re-challenge model was established. After the initial 14-day induction and treatment phase followed by a 21-day recovery period, mice were re-challenged with a reduced dose of IMQ (31.25 mg/mouse) for 7 consecutive days (**Figure 3a**). During the recovery phase, skin lesions resolved in all groups. Upon secondary IMQ challenge, mice pretreated with PBS developed pronounced erythematosquamous plaques with marked epidermal hyperplasia, whereas mice previously treated with SN exhibited milder lesions, with the most pronounced protection observed in the high-dose SN group (**Figures 3b–d**). H&E staining showed that prior SN treatment reduced relapse-associated epidermal thickening, and serum levels of TNF-α, IL-17A, and IFN-γ were also significantly decreased (**Figures 3e–i**).

### Transcriptional profiles of SN-treated mice

**Figure 4:**
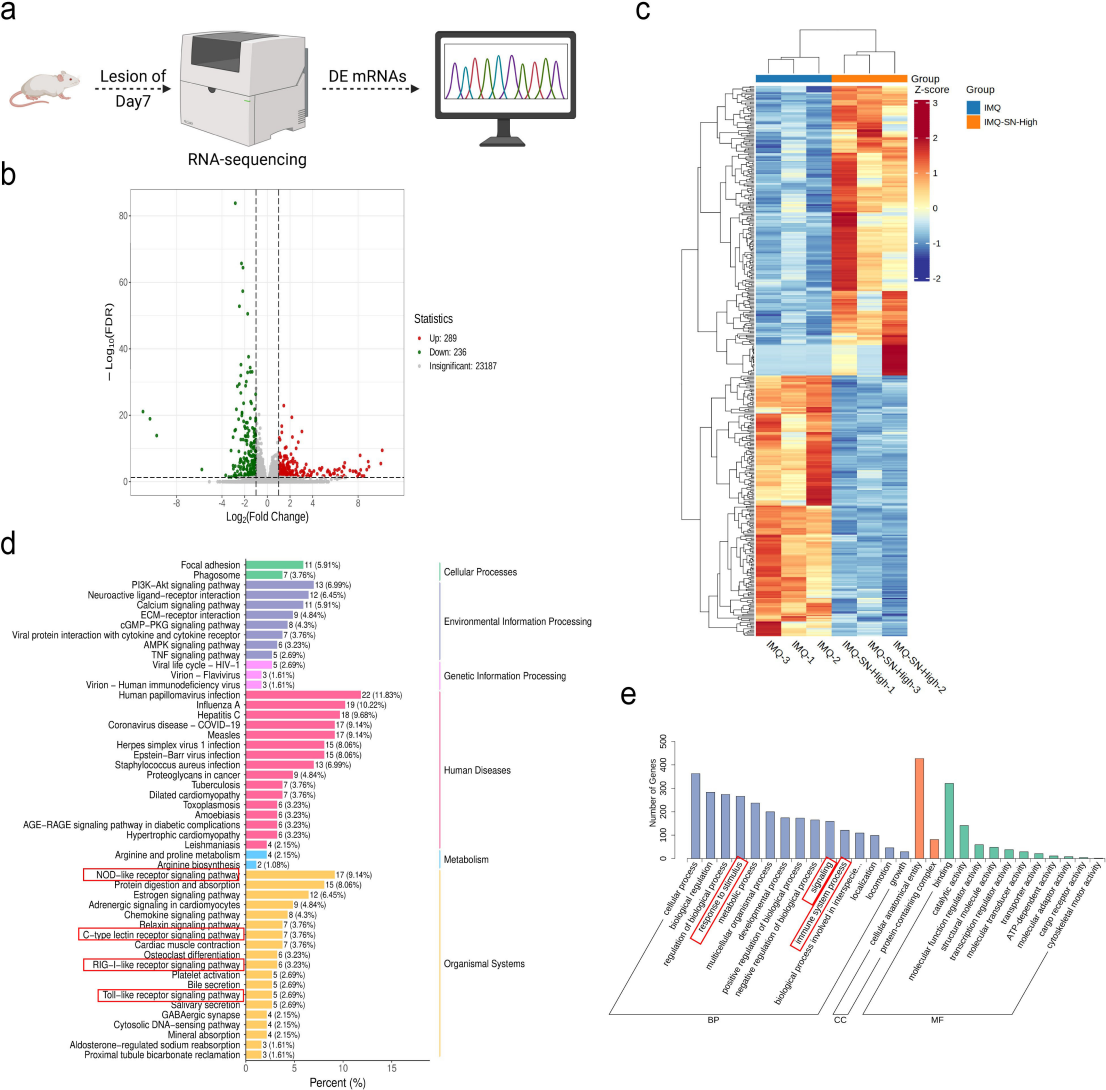
The analysis of DEGs. **(a)** Schematic illustration of transcriptome sequencing analysis; **(b)** Cluster heatmap of DEGs; **(c)** Volcano plot of DEGs; **(d, e)** KEGG and GO analyses of DEGs. The boxed pathways in **(d, e)** highlight the principal PRR-related signaling pathways altered by SN treatment, with the NLR signaling pathway showing the most prominent enrichment.

To elucidate the molecular mechanisms underlying the therapeutic effects of SN, RNA sequencing (RNA-seq; n=4) was performed on dorsal skin samples from the Control, IMQ, and IMQ-SN-High groups (**Figure 4a**). Differential expression analysis identified 4,670 differentially expressed genes (DEGs) between the Control and IMQ groups, including 2,111 upregulated and 2,559 downregulated genes. In comparison, 525 DEGs were detected between the IMQ and IMQ-SN-High groups, of which 289 were upregulated and 236 were downregulated (**Figures 4b, c**).

Gene Ontology (GO) and Kyoto Encyclopedia of Genes and Genomes (KEGG) pathway enrichment analyses of the DEGs between the IMQ and IMQ-SN-High groups revealed significant enrichment in pattern recognition receptor (PRR)-related signaling pathways, including NOD-like receptors (NLRs), Toll-like receptors (TLRs), C-type lectin receptors (CLRs), and RIG-I-like receptors (RLRs). Notably, the NLR signaling pathway exhibited both the highest number of enriched genes and the greatest statistical significance among these pathways (**Figures 4d, e**), suggesting that SN may exert its therapeutic effects in psoriasis predominantly through modulation of NLR-mediated signaling.

### SN inhibits NLRP3 inflammasome activation

**Figure 5:**
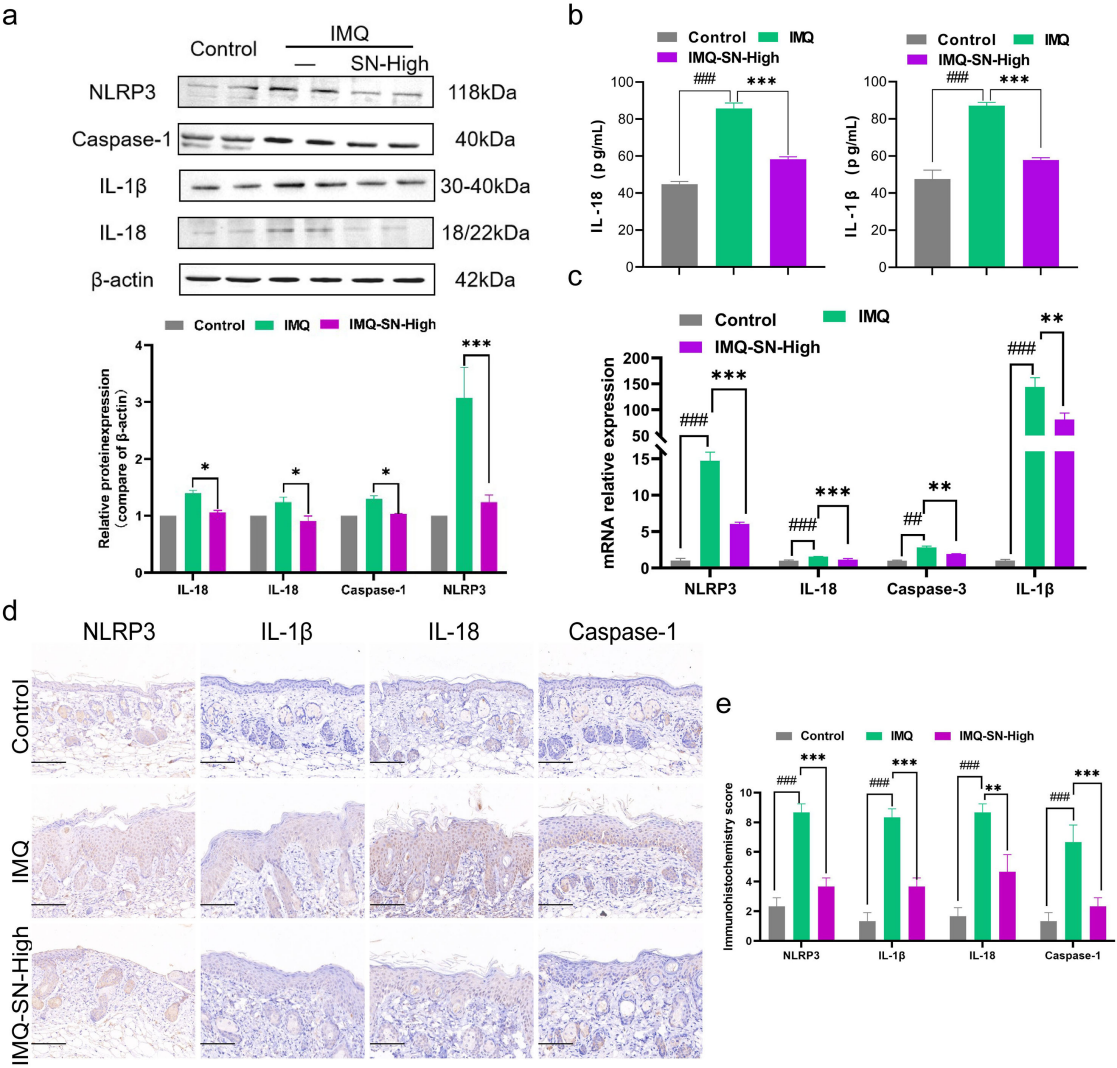
SN inhibited the activation of NLRP3 inflammasome in the psoriasis-like mouse model. **(a)** The relative protein expression of NLRP3 inflammasome was detected using WB; **(b)** IL-18 and IL-1β levels in serum were analyzed by ELISA; **(c)** qRT-PCR analysis of mRNA expression levels of NLRP3 inflammasome; **(d)** The relative protein expression of NLRP3 inflammasome was analyzed by IHC (×200). **(e)** Immunohistochemistry score for relative NLRP3 inflammasome protein expression. The data are expressed as mean ± SD (n=4). #p < 0.05, ##p < 0.01, and ###p < 0.001 vs. the control group; *p < 0.05, **p < 0.01, and ***p < 0.001 vs. the IMQ group. Scale bar = 100 μm.

Given the enrichment of NLR-related signaling pathways observed in the transcriptomic analysis, we further examined the activation status of the NLRP3 inflammasome. Serum analysis of IMQ-induced psoriasiform mice showed significantly elevated levels of IL-1β and IL-18 compared with control mice; these increases were markedly attenuated following SN treatment (**Figure 5b**). Furthermore, Western blotting, quantitative RT-PCR, and immunohistochemical analyses consistently demonstrated enhanced activation of the NLRP3 inflammasome in psoriatic lesions, whereas SN treatment effectively suppressed NLRP3 expression and the associated inflammatory mediators (**Figures 5a, c–e**).

### Chemical profiling of SN by UPLC/MS

**Figure 6:**
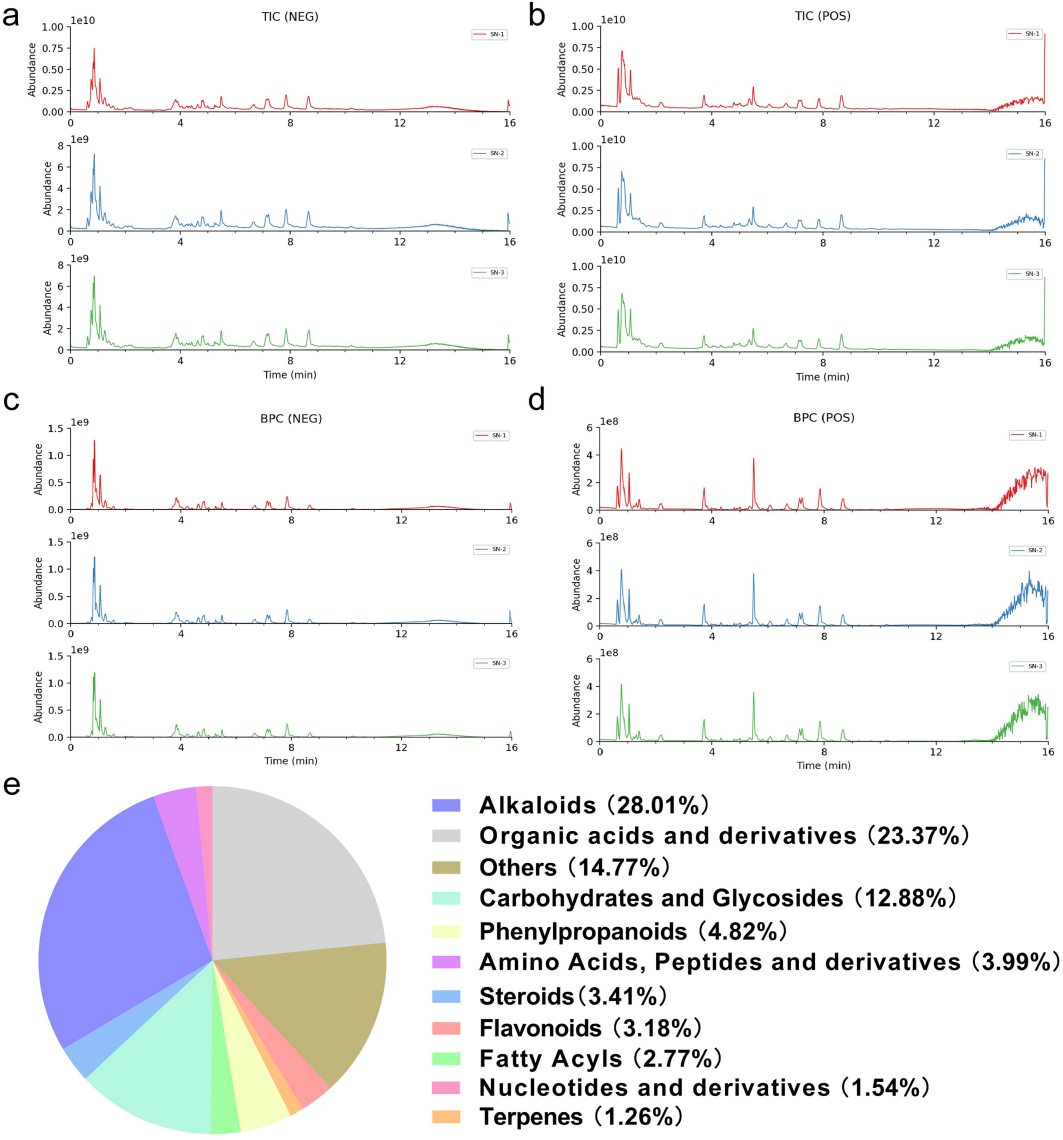
Analysis of main constituents of SN by UPLC/MS. **(a, b)** (TIC of SN, **(a)** Negative ion mode, **(b)** Positive ion mode; **(c, d)** BPC of SN, **(c)** Negative ion mode, **(d)** Positive ion mode; **(e)** Pie chart of the types of components contained in the aqueous extract of SN.

To characterize the bioactive constituents underlying the pharmacological effects of SN, UPLC/MS analysis was performed on its aqueous extract. The total ion chromatograms (TICs) are shown in **Figures 6a, b**, while the base peak chromatograms (BPCs) acquired in positive and negative ion modes are presented in **Figures 6c, d**. In total, 570 compounds were identified. The chemical composition was predominantly composed of alkaloids (28.01%), followed by organic acids and derivatives (23.37%), sugars and glycosides (12.88%), phenylpropanoids (4.84%), amino acids and peptides (3.99%), fatty acyls (2.77%), terpenoids (1.26%), nucleotides and derivatives (1.54%), flavonoids (3.18%), steroids (3.41%), and other constituents (14.77%)(**Figure 6e**). Notably, alkaloids constituted the largest proportion of detected compounds, drawing particular attention. Consistent with previous reports identifying alkaloids as the principal bioactive components of SN, subsequent analyses focused on this chemical class. The top 10 alkaloids identified in SN are summarized in **Table 1**.

**Table 1:** Top 10 alkaloid-related constituents of SN identified by UPLC–MS.

No.	Formula	Metabolites	Score	m/z	Ion mode	The average ratio of peak areas(%)
1	C45H73NO15	Solamargine	49.3	868.5031	POS	8.037593008
2	C45H73NO16	Solasonine	48.5	884.4981	POS	6.318068798
3	C7H7NO2	Trigonelline	47.1	138.0549	POS	1.532742189
4	C7H13NO2	Stachydrine	41.2	144.1019	POS	0.893077754
5	C27H45NO3	Peimine	42.2	414.3357	POS	0.835452079
6	C10H13NO2	Fusaric acid	46.9	180.1017	POS	0.313050512
7	C8H13NO2	Scopine	42.3	156.1021	POS	0.193684216
8	C11H13NO6	Nicotinic acid riboside	56.4	256.0815	POS	0.146946397
9	C18H19NO5	N-Feruloyloctopamine	54.5	328.1195	NEG	0.135478013
10	C27H43NO3	Yubeinine	52.3	430.3311	POS	0.113383049

### Trigonelline as a potential active compound

**Figure 7:**
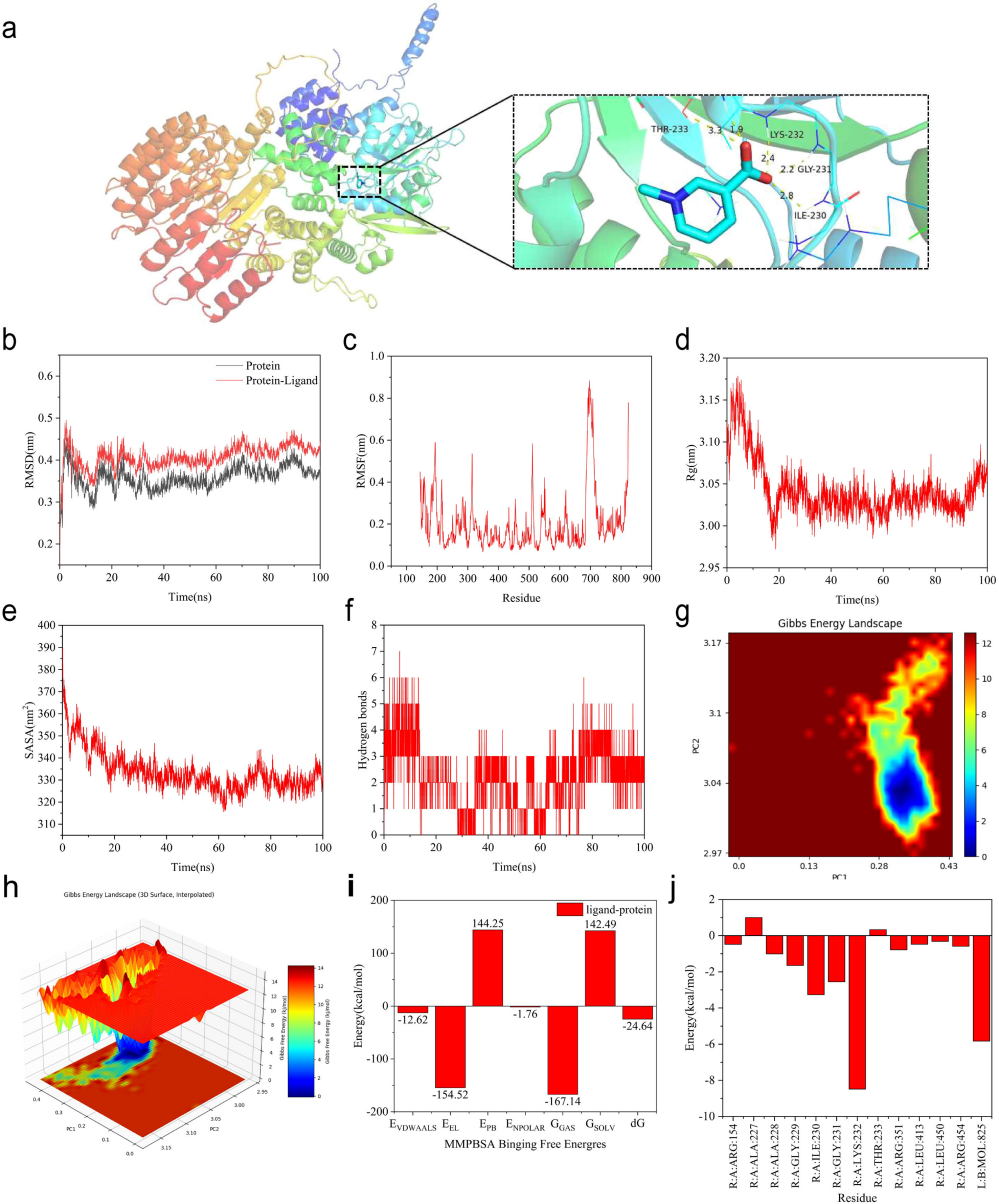
Molecular docking and molecular dynamics simulations predict the affinity and dynamics of trigonelline and NLRP3 binding. **(a)** Diagram of the Binding Mode of Trigonelline with NLRP3; **(b)** The overall structural stability was evaluated using RMSD; **(c)** Residue flexibility was analyzed through RMSF; **(d)** Molecular compactness was characterized by the Rg; **(e)** Solvent exposure is represented by the SASA; **(f)** Interaction stability was assessed based on the number of hydrogen bonds formed between the protein and ligand; **(g-j)** Complex stability and binding affinity were examined through free energy topography in both 2D and 3D representations, as well as mmPBSA analysis, which includes mean binding free energy and residue decomposition energy.

Among the top five alkaloid-related constituents identified in SN, trigonelline showed the most pronounced inhibition of cytokine-stimulated keratinocyte proliferation *in vitro* and the greatest efficacy in the IMQ-induced psoriasis-like model (**Supplementary Figures S1**, **S2**). Molecular docking analysis demonstrated that trigonelline binds to NLRP3 with a binding energy of −5.4 kcal·mol⁻¹, exceeding the commonly accepted threshold for stable ligand–target interactions (**Figure 7a**, **Table 2**). To further evaluate the stability of this interaction, molecular dynamics simulations were conducted. The NLRP3–trigonelline complex reached equilibrium within 10 ns, with the ligand remaining stably positioned within the binding pocket throughout the simulation. Ligand binding induced a more compact protein conformation, indicative of enhanced structural stability, and hydrogen bonding interactions contributed substantially to complex stabilization. Binding free energy analysis over the 0–100 ns simulation period revealed a mean value of −24.64 kcal/mol. Residue decomposition analysis identified LYS-232, ILE-230, and GLY-231 as key contributors to the interaction (**Figures 7b-j**). Collectively, these results support trigonelline as a principal bioactive constituent of SN and suggest that its anti-psoriatic effects may be mediated, at least in part, through direct interaction with NLRP3.

**Table 2:** Predicted binding energies between trigonelline and NLRP3 from molecular docking.

Mode	Affinity (kcal/mol)	Dist from best mode rmsd l.b. rmsd u.b.	
		rmsd l.b.	rmsd u.b.
1	-5.4	0.000	0.000
2	-5.3	9.946	10.743
3	-5.1	9.455	11.068
4	-4.9	9.177	10.351
5	-4.8	37.318	38.012
6	-4.7	9.827	11.033
7	-4.5	16.600	17.154
8	-4.5	7.902	9.438
9	-4.3	1.810	2.369
10	-4.2	7.026	8.596
11	-4.1	20.549	21.319
12	-3.9	25.753	26.500
13	-3.9	34.963	35.850
14	-3.9	25.588	26.152
15	-3.9	19.676	20.566
16	-3.9	34.590	35.892
17	-3.9	29.567	30.839
18	-3.7	34.498	35.800
19	-3.7	24.871	25.517
20	-3.7	34.856	36.070

## Discussion

Pattern recognition receptors (PRRs) contribute to the initiation and amplification of inflammatory responses in psoriasis, acting upstream of canonical adaptive immune circuits such as the IL-23/Th17 axis (18). Among PRR-driven pathways, the NLRP3 inflammasome functions as a key innate immune hub that promotes maturation of IL-1β and IL-18 and can thereby amplify Th17 responses and sustain keratinocyte hyperproliferation (19). Aberrant NLRP3 activation has been implicated not only in psoriasis but also in other chronic inflammatory diseases, including diabetes, gout, and atherosclerosis (20–22), highlighting NLRP3 as a convergent inflammatory target.

In this study, we combined public transcriptomic analyses, clinical biopsy validation, and mechanistic interrogation in primary and relapse IMQ-induced models to establish that the NLRP3 inflammasome is activated in psoriatic inflammation and is suppressed by SN. Notably, although CASP1 transcript levels were not significantly altered in the public datasets, protein-level expression and downstream cytokine readouts (IL-1β/IL-18) were consistently increased in clinical and murine lesions. This discrepancy is biologically plausible because inflammasome activation is primarily regulated by post-translational processing and Caspase-1 cleavage rather than mRNA abundance.

Mechanistically, suppression of NLRP3 inflammasome signaling may influence broader immune networks relevant to psoriasis. IL-1β can promote expansion and activation of IL-17-producing T cells, while IL-18 synergizes with IL-23 to strengthen inflammatory circuits that drive epidermal hyperplasia (23, 24). Accordingly, inhibition of NLRP3-dependent cytokine maturation provides a coherent framework linking SN treatment to attenuation of the IL-23/Th17–keratinocyte feed-forward loop and reduced systemic inflammatory cytokines observed *in vivo*.

Consistent with this framework, SN treatment alleviated erythema, scaling, and thickening in the primary IMQ model and reduced keratinocyte proliferation markers (Ki-67 and PCNA), accompanied by decreased circulating TNF-α, IL-17A, and IFN-γ. Importantly, SN also mitigated disease recurrence in the relapse model, improving histopathological features such as acanthosis, parakeratosis, and dermal inflammatory infiltration. While repeated IMQ exposure may introduce residual or carryover effects, we incorporated a 21-day recovery phase to allow macroscopic lesion resolution prior to re-challenge, and all groups underwent identical induction and re-challenge schedules, reducing systematic bias.

Re-challenge models in psoriasis are often discussed in the context of immune memory; however, the present study was not designed to distinguish durable pharmacodynamic inhibition from modulation of trained immunity or other memory-like inflammatory programs. Therefore, the attenuated response observed after prior SN treatment should be interpreted cautiously as sustained protection upon secondary IMQ challenge. This effect may reflect prolonged suppression of inflammatory pathways after the primary treatment phase, modulation of trained innate immunity, or a combination of both. Future studies incorporating washout-controlled designs, assessment of resident memory T cells, and analyses of trained-immunity-associated myeloid reprogramming will be needed to clarify the mechanistic basis of this re-challenge phenotype.

SN has a long-standing history in traditional Chinese medicine for inflammatory dermatoses. Clinically, SN is a principal herb of Longkui Yinxiao formulations used at Shanghai Skin Disease Hospital, and previous reports suggest that related formulations can modulate inflammatory signaling pathways in psoriasiform models (16). Compared with many candidate anti-psoriatic plants, the established clinical utilization and alkaloid-rich chemical profile of SN facilitate mechanistic deconvolution and quality-control development, which are essential for natural-product drug development.

Because herbal extracts contain multiple constituents, we performed UPLC–MS profiling and prioritized alkaloids as a dominant chemical class. Among the major alkaloids, trigonelline showed the most prominent inhibitory effect on inflammatory keratinocyte proliferation and demonstrated therapeutic benefit *in vivo* (**Supplementary Figure S1**). Molecular docking and molecular dynamics simulations supported a stable interaction between trigonelline and NLRP3. Nevertheless, trigonelline is known to have pleiotropic bioactivities, and its contribution to anti-psoriatic effects may involve additional targets beyond NLRP3. Future studies using genetic or pharmacological NLRP3 perturbation, direct inflammasome activity assays (e.g., Caspase-1 cleavage, ASC speck formation, IL-1β processing), and purified-compound pharmacokinetics will be important to further substantiate target engagement.

From a translational perspective, current psoriasis therapies (e.g., biologics targeting TNF-α, IL-17, or IL-23) are highly effective but can be limited by cost, injection burden, and relapse after discontinuation. Targeting innate immune nodes such as NLRP3 may provide a complementary strategy, potentially serving as an adjunct to existing regimens or as a candidate for topical/oral small-molecule development. The low cost and accessibility of SN, together with identification of trigonelline as a candidate marker compound, support future efforts toward standardization and clinical evaluation.

Several limitations should be acknowledged. First, sample sizes were modest in animal experiments, although consistent effects were observed across multiple readouts and models. Second, our study focused on NLRP3 inflammasome-related endpoints and did not comprehensively profile all immune cell subsets in skin. Third, UPLC–MS annotation provides putative identification for many components, and additional targeted quantification and validation are warranted for quality control. Despite these limitations, our multi-layer evidence supports NLRP3 inflammasome inhibition as a central mechanism of SN and provides a prioritized active constituent for follow-up development.

## Conclusion

In conclusion, aberrant activation of the NLRP3 inflammasome plays a pivotal role in the development and recurrence of psoriasis. SN effectively alleviates psoriasis-like skin inflammation and suppresses disease relapse by inhibiting NLRP3 inflammasome activation and downstream inflammatory cytokine production. Furthermore, trigonelline was identified as a key active constituent of SN with potential NLRP3-targeting activity. These findings provide mechanistic and pharmacological evidence supporting the therapeutic potential of SN in psoriasis and highlight NLRP3 inflammasome inhibition as a promising strategy for the treatment of chronic inflammatory skin diseases.

## Data Availability

The data presented in this study are deposited in the NCBI BioProject repositories. accession number PRJNA1453987.
