# Supplementary material for: Mechanisms and active components of Solanum nigrum in the amelioration of psoriatic lesions
Source: Front Immunol. 2026 Apr 24;17:1801799. doi: 10.3389/fimmu.2026.1801799 (PMC13152748; doi:10.3389/fimmu.2026.1801799)
Supplement: Supplementary file 1 [file DataSheet1.docx]

**Supplementary Table S1. Animal groups and interventions**

| **Model** | **Group** | **IMQ regimen** | **Intervention** | **Dose/route** | **n** |
| --- | --- | --- | --- | --- | --- |
| Primary model | Control | None | PBS | 200 µL, oral gavage | 4 |
| Primary model | Vehicle-IMQ | 5% IMQ cream daily ×7 d (62.5 mg/mouse/day) | PBS | 200 µL, oral gavage | 4 |
| Primary model | IMQ + SN-Low | 5% IMQ cream daily ×7 d | SN extract | 2.16 g/kg/day, oral gavage | 4 |
| Primary model | IMQ + SN-High | 5% IMQ cream daily ×7 d | SN extract | 8.65 g/kg/day, oral gavage | 4 |
| Primary model | IMQ + MTX | 5% IMQ cream daily ×7 d | MTX | 1 mg/kg, oral gavage | 4 |
| Relapse model | Non-relapse | Primary induction + 21 d recovery; no re-challenge | None |  | 4 |
| Relapse model | Relapse-IMQ | Re-challenge IMQ ×7 d (31.25 mg/mouse/day) | None |  | 4 |
| Relapse model | Relapse + SN-Low | Re-challenge IMQ ×7 d | None |  | 4 |
| Relapse model | Relapse + SN-High | Re-challenge IMQ ×7 d | None |  | 4 |

**The dosage calculation method for SN**

If an adult weighing 60 kilograms takes 16 tablets of Longkui Yinxiao Tablets daily, with each tablet containing 0.9g of raw SN (Longkui), then the daily dosage of Longkui for an adult is 900/1000*16=14.4 g; 14.4 g/60 kg = 0.24 g/kg (for species A animal). Based on the dose conversion coefficient table for animals and humans and the formula "dose of Animal B (mg/kg) =W* Dose of Animal A (mg/kg)", the daily intake of SN for mice of animal B is 9.01×0.24=2.1624 g/kg (= 43.248 mg per 20 g mouse).

**The scoring details of Immunohistochemistry staining**

Immunohistochemistry was evaluated using a semi-quantitative scoring system based on staining intensity and the proportion of positive cells. Staining intensity was scored as 0 (no staining), 1 (light yellow), 2 (brownish yellow), and 3 (dark brown). The proportion of positive cells was scored as 0 (<5%), 1 (5–25%), 2 (26–50%), 3 (51–75%), and 4 (>75%). The final IHC score was calculated as intensity × proportion (range 0–12) and categorized as negative (0), weakly positive (1–4), moderately positive (5–8), or strongly positive (9–12). Two investigators blinded to group allocation independently scored all sections, and the mean score was used for statistical analysis.

**Supplementary Table S2: Primers used for qPCR assays**

| **Gene** | **Forward primer** | **Reverse primer** |
| --- | --- | --- |
| GAPDH | TGGGAAGCTGTGGCGTGATG | TCAGATCCACAACCGACACATT |
| NLRP3 | CCTGGGGGACTTTGGAATCA | GATCCTGACAACACGCGGA |
| IL-1β | TGCCACCTTTTGACAGTGATG | TGATGTGCTGCTGCGAGATT |
| IL-18 | GCCTGTGTTCGAGGATATGACT | CCTTCACAGAGAGGGTCACAG |
| Caspase-1 | GAAAGACAAGCCCAAGGTTA | GGTGTTGAAGAGCAGAAAGC |

**Effects of major alkaloids from SN on keratinocyte proliferation in vitro**

Based on UPLC–MS profiling, five abundant alkaloid-related constituents in *Solanum nigrum* (SN) were prioritized (solasonine, solamargine, trigonelline, stachydrine, and peimine). Concentration ranges were selected according to the literature and/or preliminary cytotoxicity testing. Stachydrine and peimine showed no significant effect on keratinocyte proliferation (P>0.05), whereas solasonine, solamargine, and trigonelline significantly inhibited cytokine-stimulated keratinocyte hyperproliferation (P<0.05–0.01).


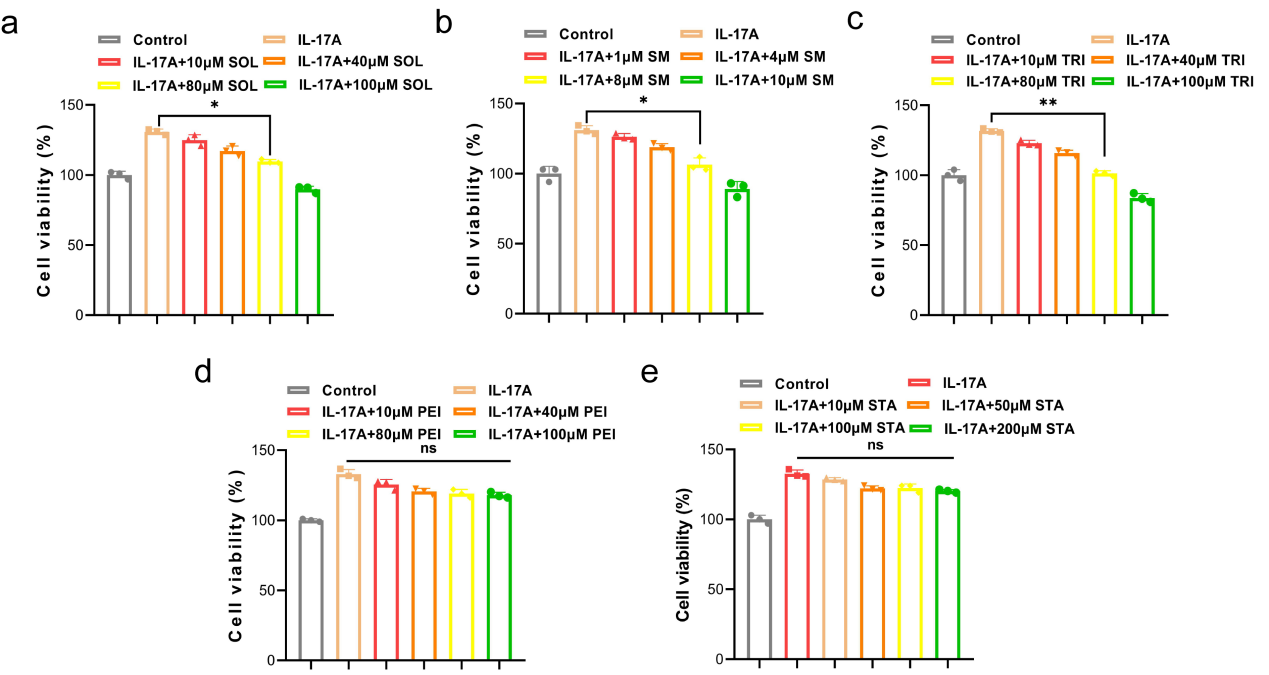


Supplementary Fig. S1. Effects of five SN constituents on keratinocyte proliferation in vitro. (a-e) Keratinocytes were stimulated under an inflammatory condition and treated with solasonine (a, SOL), solamargine (b, SM), trigonelline (c, TRI), peimine (d, PEI), or stachydrine (e, STA) at the indicated concentrations. Cell proliferation/viability was assessed by CCK-8. Data are presented as mean ± SD (n = 3). ns, not significant; *P<0.05, **P<0.01.

**In Vivo Pharmacodynamic Evaluation of Three Alkaloid Active Ingredients**

Based on a comprehensive review of the relevant literature, both low and high concentrations were established for solasonine, solamargine, and trigonelline. Supplementary Fig. S2a illustrates that mice treated with IMQ exhibited significant erythema and scaling on their skin; however, in the treatment groups, the high-dose trigonelline group demonstrated the most notable improvement in dorsal skin lesions (P<0.001). Supplementary Fig. S2b, c, and d further indicate that the high-dose trigonelline group significantly reduced the PASI score (P<0.001) as well as epidermal thickness in psoriasis-like mice. These findings suggest that trigonelline effectively inhibits abnormal epidermal proliferation and reduces epidermal thickness. Collectively, these experimental results underscore the potential of trigonelline as a therapeutic agent for psoriasis.


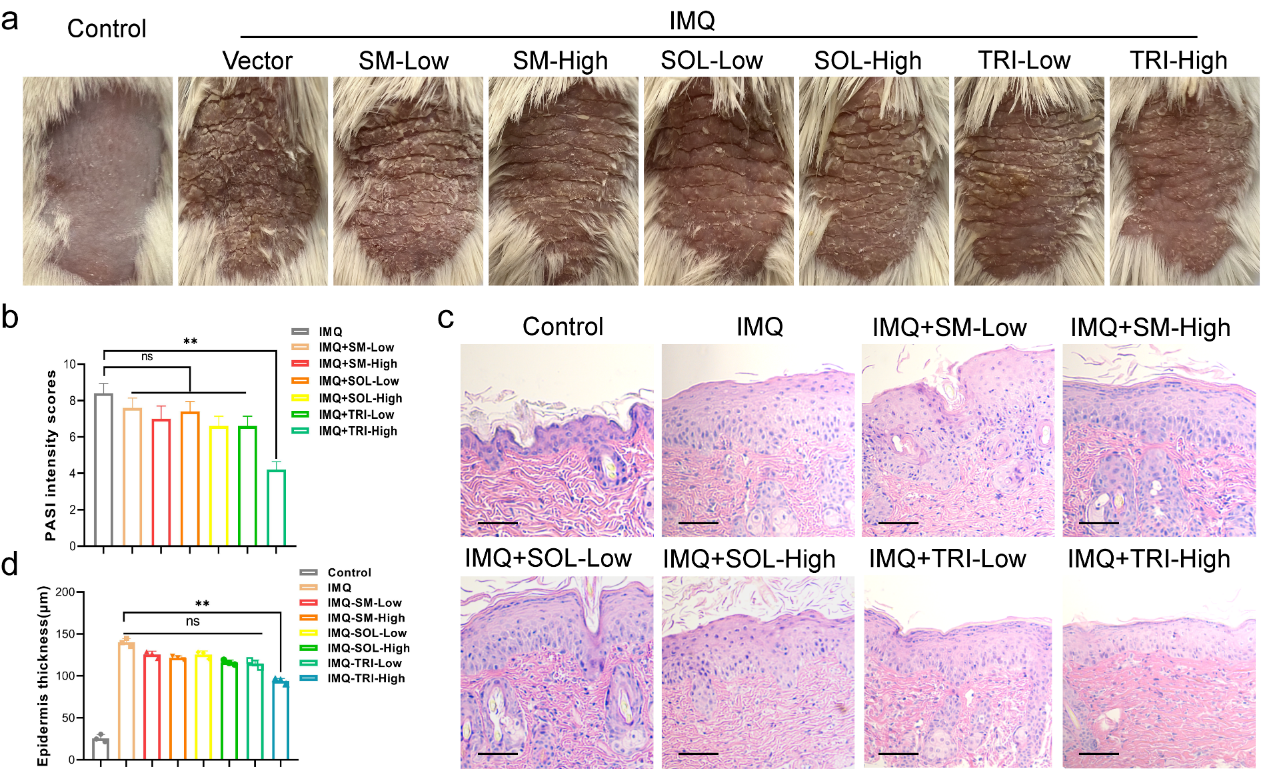


Supplementary Fig. S2. In vivo effects of three SN alkaloids in IMQ-induced psoriasis-like mice. (a) Representative dorsal skin images after treatment with solasonine (SOL), solamargine (SM), or trigonelline (TRI) at the indicated doses. (b) PASI scores. (c) Representative H&E images (×200). Scale bar = 100 μm**.** (d) Quantification of epidermal thickness. Data are presented as mean ± SD (n = 4). ns, not significant; **P<0.01.

**Supplementary Methods: Modified PASI scoring in mice**

Erythema, scaling, and thickness of the dorsal skin were each scored daily on a 0–4 scale (0 = none, 1 = slight, 2 = moderate, 3 = marked, 4 = very marked). The cumulative PASI score ranged from 0 to 12 per mouse. Scoring was performed by two investigators blinded to group allocation, and the mean score was used for analysis.

**Supplementary Methods: GEO microarray analysis parameters**

Datasets GSE13355 and GSE14905 were analyzed in R. Expression matrices were log2-transformed when needed. Differential expression was assessed using limma with Benjamini–Hochberg FDR correction. Unless otherwise stated, FDR-adjusted p < 0.05 was used as the significance threshold; for exploratory DEG lists, an additional |log2 fold change| ≥ 1 threshold was applied. For genes with multiple probes, the probe with the highest average expression was retained.

**Supplementary Methods: UPLC–MS sample preparation and conditions**

For LC–MS, 100 μL SN extract was mixed with 1 mL methanol containing an internal standard (4 μg/mL), vortexed for 1 min, ultrasonicated in an ice-water bath for 60 min, incubated at −40 °C for 30 min, and centrifuged (12,000 rpm, 10 min, 4 °C). The supernatant was diluted 10-fold with water containing the internal standard (4 μg/mL), and 200 μL was transferred to LC–MS vials. Pooled QC samples were prepared by combining equal aliquots and used to monitor system stability.

Chromatography and MS acquisition were performed on ACQUITY UPLC I-Class HF (Waters) coupled to Q Exactive Orbitrap (Thermo). Column: ACQUITY UPLC HSS T3 (100 mm × 2.1 mm, 1.8 μm); column temperature 45 °C; flow rate 0.35 mL/min; injection 5 μL; mobile phase A (0.1% formic acid in water) and B (acetonitrile). Gradient: 0–2 min, 95% A; 2–4 min, 95–70% A; 4–8 min, 70–50% A; 8–10 min, 50–20% A; 10–14 min, 20–0% A; 14–15 min, 0% A; 15.1–16 min, 95% A. MS: HESI, positive/negative modes; spray voltage 3800/−3000 V; capillary 320 °C; aux heater 350 °C; sheath/aux gas 35/8; S-lens RF 50; scan range m/z 100–1200; full MS resolution 70,000; MS/MS resolution 17,500; DDA Top 8; stepped NCE 10/20/40. Data processing and annotation were performed using Progenesis QI v3.0 with database matching and manual curation by EIC and MS/MS interpretation.
